# Acceptability and feasibility of a mobile electronic medical record system for community-based antiretroviral therapy in Lilongwe, Malawi: A rapid qualitative analysis

**DOI:** 10.1371/journal.pone.0303416

**Published:** 2025-05-23

**Authors:** Christine Kiruthu-Kamamia, Astrid Berner-Rodoreda, Gillian O’Bryan, Odala Sande, Jacqueline Huwa, Agnes Thawani, Hannock Tweya, Wim Groot, Milena Pavlova, Caryl Feldacker

**Affiliations:** 1 United Nations University – Maastricht Economic and Social Research Institute on Innovation and Technology, Maastricht, Netherlands; 2 Lighthouse Trust, Lilongwe, Malawi; 3 International Training and Education Center for Health, Seattle, Washington, United States of America; 4 Heidelberg University, Heidelberg, Germany; 5 Department of Global Health, University of Washington, Seattle, Washington, United States of America; 6 Department of Health Services Research, CAPHRI, Faculty of Health, Medicine and Life Sciences, Maastricht University Medical Center, Maastricht University, Maastricht, The Netherlands; Khoy University of Medical Sciences, ISLAMIC REPUBLIC OF IRAN

## Abstract

**Background:**

Mobile health (mHealth) is reshaping healthcare delivery, especially in HIV management. The World Health Organization advocates for mHealth to provide healthcare workers (HCWs) with real-time data, enhancing client care. However, in Malawi’s Lighthouse Trust antiretroviral therapy (ART) clinic, the nurse-led community-based ART (NCAP) program faces hurdles with data management due to lack of access to electronic medical records systems (EMRS) in the community setting. EMRS are not typically available in differentiated service delivery settings where reliable power and internet are often unavailable. We used human-centered design (HCD) processes to create a mobile EMRS prototype, the Community-based ART Retention and Suppression (CARES) app. CARES aims to simplify workflow for HCWs and improve client care. As part of prototype testing, we explore CARES’ feasibility and acceptability from the perspective of NCAP HCWs.

**Methods:**

We conducted in-depth interviews among 15 NCAP HCWs. We used a rapid qualitative analysis approach guided by the Technology Acceptance Models (TAM1 and TAM2). The study complied with the Consolidated Criteria for Reporting Qualitative Research (COREQ).

**Results:**

HCWs demonstrated high perceived usefulness for the CARES app to improve healthcare delivery and data management, both of which could facilitate continued and effective use. However, challenges such as app performance, data integration, and system navigation were significant barriers to ease of use, reducing acceptance and current feasibility of scale-up. Despite challenges, HCWs remained optimistic about CARES’ potential to enhance NCAP clinical decision-making and data flow in future, likely reflecting organizational expectations for CARES optimization. Beyond the TAM framework, additional themes of consistent staff engagement and considerations for sustainability were identified as critical factors for long-term success. HCWs emphasized the need for continuous training and stakeholder engagement, improved infrastructure, data security protections, and establishing the CARES app and EMRS integration to facilitate CARES’ sustained expansion.

**Conclusion:**

The study findings underscore the importance of HCD for mHealth buy-in. As HCWs were invested in CARES success, they remained optimistic that the app could enhance NCAP services if user experience and app performance improved. Incorporation of HCW feedback could help deliver the promise of CARES, current and future.

## Introduction

Mobile health (mHealth), the use of mobile devices to improve healthcare delivery, has become a pivotal force of change in healthcare, including for HIV management [1–4]. Recognizing the potential of technological advancements, the World Health Organization (WHO) emphasizes using innovative mHealth technologies to optimize person-centered service delivery. These technologies ensure that healthcare workers (HCWs) have the necessary data at the point of care [5]. In low-resource settings, mHealth innovations have been shown to improve HIV treatment outcomes. These include enhanced antiretroviral treatment (ART) adherence, retention, and viral load suppression [6–8].

HIV remains a pervasive global issue, particularly in sub-Saharan Africa, with countries like Malawi experiencing some of the world’s highest HIV prevalence rates. The WHO advocates the adoption of differentiated service delivery (DSD) models in HIV care. A DSD model is a client-centered approach providing tailored care to people living with HIV often based on specific client characteristics including age, socioeconomic status, background, viral load levels, and stability on ART. DSD approaches often include outreach activities to lower the burden on both clients and the health system [9]. Community-based DSD models shift stable ART clients from crowded clinics to care provided in the community for enhanced accessibility and convenience for clients [9,10].

Lighthouse Trust is the largest ART provider in Malawi that cares for over 65,000 clients alive on ART as part of Malawi Ministry of Health (MoH) public service delivery [11,12]. In 2016, the nurse-led community ART program (NCAP) was piloted at the Lighthouse Trust ART clinic in Malawi; NCAP has been growing successfully with client, clinician and MoH support [13]. Distributing ART in the community enables clients to refill ART closer to their homes through mobile clinics or community meeting points. This approach reduces travel times for the client and decongests healthcare facilities [10].

Providing quality care requires a robust, comprehensive monitoring and evaluation (M&E) system [14,15]. This is often more complex, but critical in a community ART setting, where ART is distributed within the community. In community-based DSD, HCWs typically rely on paper-based reporting, which can increase the risk of loss-to-follow-up and impede viral load (VL) monitoring [16–18]. After NCAP was piloted, workers and clients responded favorably, and clients preferred receiving ART in the community-based DSD model rather than in a facility. However, workers found NCAP M&E challenges due to the lack of an electronic medical record system (EMRS), which eases HCW workload and streamlines reporting [13]. In Malawi, the EMRS was developed to overcome the lack of reliable data and inadequate use of data in healthcare planning [19]. To date, EMRS is only available in static hospitals or health facilities with stable connectivity. Community settings lack such infrastructure [19]. This poses a critical challenge to EMRS expansion in Malawi. mHealth can be used to fill this gap by connecting with the EMRS system to strengthen ART client care and management. Interoperating mHealth with EMRS holds promise to expand EMRS reach, thereby streamlining data capture, management, and analytics to enhance efficiency [20–22]. Interoperability may also help ensure continuous client monitoring and support, regardless of location, leading to more informed decision-making and improved care outcomes.

Lighthouse Trust, the International Training and Education Centre for Health, and Medic collaborated to develop a tablet-based, offline-first, mHealth app to mirror the EMRS that could operate in NCAP settings: the Community-based ART Retention and Suppression (CARES) app. CARES emulates the functionality and features of the EMRS with built-in prompts and alerts, ensuring compliance with national ART guidelines and enhancing the quality of client care. We used participatory human-centered design (HCD) processes to design the CARES app, working iteratively with NCAP workers to launch the CARES prototype [23]. For Lighthouse clients, CARES operates like EMRS, mimicking the flow and content of routine visits for clients in the clinic setting. The CARES app and the HCD process utilized for its development were detailed previously [23].

The aim of this study was to qualitatively explore the acceptability and feasibility of the CARES prototype among Lighthouse Trust ART HCWs who routinely interact with the CARES app or its data. This study took place during the CARES prototype testing and optimization phase. In-depth interviews (IDIs) with key HCWs involved in CARES aimed to identify facilitators and barriers to the use of the CARES prototype from the perspective of healthcare services delivery. Data collection and analysis were informed by the Technology Acceptance Model (TAM) information systems theory [24]. The TAM framework aims to explain user behavior regarding technology acceptance, focusing on perceived usefulness and perceived ease of use as fundamental determinants of technology adoption. Understanding the perspectives of HCW users on the CARES prototype app is essential for understanding the intervention’s challenges, opportunities, and potential feasibility to enhance ART care delivery and, ultimately, client outcomes at scale. Overall, we hope our research will inform both CARES improvements and the development and implementation of other future mobile mHealth interventions for community ART programs in Malawi and beyond. We also aim to assess the applicability of TAM, and its extensions, to developing an app for improved community-based mHealth services, hoping to inform future TAM adaptation in LMIC mHealth contexts.

## Methodology

### Design

A qualitative design, using IDIs to ascertain HCW perspectives and experience in using the CARES app. We applied the Consolidated Criteria for Reporting Qualitative Studies (COREQ) guidelines (see S1 Appendix). [25].

### Setting

Lighthouse Trust operates five centers of excellence clinics across Malawi. In urban Lilongwe, Lighthouse Trust operates two flagship clinics, with a combined >37,000 ART clients [11,12,26]. In addition, Lighthouse Trust also supports care at seven peri-urban satellite sites in Lilongwe District. Lighthouse Trust uses the MoH real-time, point-of-care EMRS to guarantee compliance with national ART guidelines, enhance client care and program outcomes, and simplify reporting across all facilities. As with other MoH clinics, the EMRS at each Lighthouse facility operates independently and does not share client data across different clinics. The NCAP services are offered to clients from Lighthouse Trust flagship clinics, Lighthouse Clinic (LH) and Martin Preuss Centre (MPC), and seven satellite health facilities in Lilongwe. To date, the CARES prototype has been utilized only for clients at LH and MPC clinics.

Since 2016, Lighthouse Trust’s NCAP community DSD has provided ART in the community to stable ART clients over 18 who have been on first or second-line treatment for at least six months and are virally suppressed within Lighthouse Trust’s catchment area [13,23]. NCAP offers monthly support group services, supplying 3–6-month ART refills and facilitating specialist referrals when necessary. Previously managed with an Open Data Kit (ODK)-based tool, data for NCAP visits were manually transferred to EMRS, a process CARES aims to streamline. CARES prototype addresses several ODK limitations, including decision support, integrated care, lab management, and reducing clerical workload.

### Study population

We conducted in-depth interviews (IDIs) of 1) HCWs who used the CARES prototype for LH and MPC clients while providing care in the community, and 2) those who worked on the technical side, managing, and processing the CARES app’s data. The IDIs were conducted from September 11 to October 25, 2023. All HCWs involved with NCAP were approached to participate in the interviews, and all agreed to participate in the study. The HCWs interviewed included NCAP nurses, their managers, and all M&E staff involved in NCAP data management, IT, and research. All the nurses provide direct services to NCAP clients in the community. The community health services (CHS) manager and deputy manager oversee the NCAP program and supervise the nurses. The data officer is responsible for manually entering NCAP data into the EMRS and downloading a list of NCAP clients who missed their appointments. The IT manager is responsible for hosting the electronic data collection tools for NCAP, designing them, and managing the data as needed. The data manager supports data extraction and reporting for the CHS team and develops NCAP data collection tools. The research intern and research manager support the implementation of the CARES project. By interviewing all NCAP-related HCWs involved in the CARES project, including both clinical supervisors and data managers, we anticipated reaching a natural point of data saturation.

#### Developing CARES using a human-centered design (HCD) approach.

Medic, I-TECH, and Lighthouse Trust collaborated as a multidisciplinary team to co-create the CARES prototype app. CARES app was created utilizing the Community Health Toolkit (CHT) [27], an open-source project developed by Medic. HCD was used to engage HCWs consistently, incorporate iterative feedback, and create contextually relevant technology. CARES design, development and core functionality, including the intended workload reduction impact [28], was described in detail previously [23]. In brief, CARES is a client management application for ART that prioritizes offline functionality and is designed for use in areas with limited internet connectivity – like NCAP. CARES aims to extend the reach of the Malawi MoH EMRS. In the static facility setting, CARES was designed to assist HCWs in gathering ARV drugs for all clients they expect to encounter in the community. In the NCAP setting, CARES aimed to benefit HCWs by offering them a mobile EMRS-like app, facilitating comprehensive client reviews, and aiding decision-making with alerts for various concerns. It also allows for the enrollment of new clients in the NCAP program. After each visit, CARES automatically syncs data to point-of-care EMRS, eliminating the need for manual data entry and ensuring efficient record-keeping.

The CARES prototype was designed using the HCD approach, with Medic developers having routine discussions with NCAP providers over 12 months to increase the CARES app’s acceptability and feasibility in the NCAP setting. This lengthy but highly participatory approach prioritized understanding of diverse end-user needs, minimizing wasted resources on irrelevant features. Users included clinicians, nurses, M&E teams, data managers, and NCAP leadership. The feedback sessions, occurring weekly or twice a week, with both theory and hands-on activities, involved developers engaging providers and collaborating closely with a diverse team to tailor CARES system requirements to the local context, with iterative improvements guided by analyzing user feedback and system usage patterns [29].

### Theoretical model

Analysis of IDIs was guided by the extended Technology Acceptance Model (TAM 2) [30]. We present the results according to the TAM2 framework, including the following constructs: perceived usefulness, perceived ease of use, and external factors that influence the behavioral intention to use new technology. TAM2 is based on the original TAM [24], which identifies two distinct attitudes that serve as predictors for adopting a new information system: perceived ease of use and usefulness [30]. TAM2 expands the TAM theory by incorporating additional constructs, such as social influence that are external to the individual but may affect a person‘s willingness to use a technology and output quality that considers the technology performance. The constructs are defined in Table 1.

**Table 1:** Definitions.

Construct	Definition
**Applied definitions**	
Feasibility	The feasibility of implementing the mHealth technology by relevant, local stakeholders [31].
Acceptability	The acceptability of the technology, including its implementation, for HCWs [31].
**TAM 1 and TAM 2**	
Perceived usefulness	“The degree to which a person believes that using a particular system would enhance his or her job performance [24].
Perceived ease of use	“The degree to which an individual believes that using a particular system would be free of physical and mental effort [24].”
**TAM 2**	
Output quality	The performance of the technology or system [30].
Social influence processes	An assessment of the relationships among social factors (subjective norm, voluntariness and perceived image) to implement the technology [30].
Cognitive instrumental processes	An assessment of how individuals evaluate how well a system or technology is relevant to their job [30].
**New concepts beyond TAM constructs**	
Continuous engagement	Continuously engaging key stakeholders for technology adoption.
Sustainability	The longevity and the continuous manifestation of the benefits and outcomes of digital innovations for health workers, the standard of healthcare, and client experience long after the end of the program.

**Fig 1:**
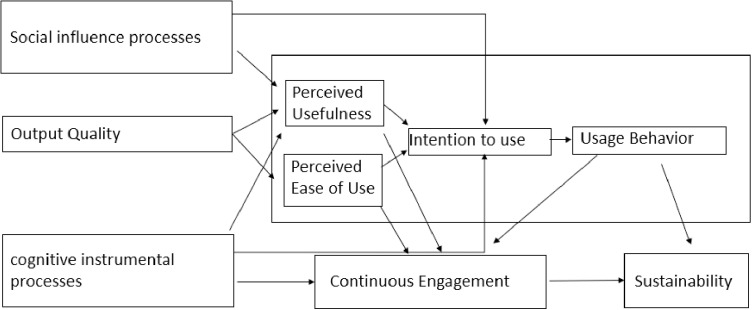
Expanded TAM2 Theory.

We modified and expanded the original TAM2 framework (Fig 1) to introduce the additional themes of: a) continuous engagement; and b) sustainability. The constructs and definitions of key terms from TAM, TAM2, and our modified model are outlined in Table 1.

### Data collection

HCWs took part in IDIs after 18 months of iterative CARES development and three months of NCAP field testing to optimize the CARES prototype. Data was collected at the LH clinic. The interview guide was informed by the TAM 2 model and CARES optimization processes. Interviews were conducted to assess participant experiences with the CARES app development and implementation process, explore benefits and challenges, and identify recommendations for improvement before the broader launch of the CARES approach. All interviews were run by an external, experienced qualitative female researcher using a semi-structured interview guide (see S2 Appendix). The interviewer was an employee of the LH clinic, but was not involved with CARES. IDIs were conducted in a private space in English and recorded and transcribed verbatim. The interview guide was pilot-tested with the first participant and revised for clarity before further IDI implementation.

### Data analysis

We used rapid qualitative analysis (RQA) to analyze the interviews. The RQA approach streamlines the qualitative data analysis process, facilitating quicker implementation and dissemination of evidence-based innovations to reduce delays in translating research findings into practical applications [32]. Instead of coding, the RQA approach consists of condensing interview transcripts into summary templates, utilizing data reduction techniques tailored for rapid turnaround in qualitative health services research. These summary templates were structured around domains aligned with the interview questions and the underlying theoretical model [33]. Two researchers developed the domains, compared and adjusted domain terms, and tested the RQA template on the first three transcripts before condensing all transcripts into one page template summaries [34]. The RQA summaries were then transferred to a matrix that provided an overview across data elements and domains. The author team analyzed the matrix in a series of meetings. Insights were discussed until a consensus was reached on the themes and sub-themes.

#### Ethics.

The study protocol was approved by the Malawi National Health Sciences Research Committee (#21/11/2830) and the Ethics Review Board of the University of Washington, Seattle, USA (STUDY00013936). All participants provided written voluntary informed consent.

## Results

Results on CARES feasibility and acceptability are organized by TAM1 (perceived usefulness and perceived ease of use) as well as by TAM 2 theme (social influence process, output quality and cognitive instrumental process) and expanded to describe concepts discovered by RQA that were not included in the original TAM 1 or TAM 2, specifically the need for continuous engagement and sustainability.

### IDI participants

We interviewed 15 HCWs involved with CARES service delivery or M&E (Table 2). The majority were male (N = 9) and nurses (N = 10). The median age of the participants was 39, with the median duration of employment at Lighthouse Trust and involvement with the NCAP being four years.

**Table 2:** Demographic characteristics.

Cadre	Female (N)	Male (N)	Median Age, years (IQR)	Median duration employed at Lighthouse, years (IQR)	Median duration involved with NCAP, years (IQR)
M&E	2	3	36(32–39)	3(2–8)	3(2–5)
Nurse	4	6	39(32–44)	5(3–14)	5(3–7)
**Total**	**6**	**9**	39(32–43)	4(2–12)	4(2–7)

IQR= interquartile range; NCAP=Nurse-led community ART

### Perceived usefulness of CARES prototype (TAM1/ TAM2)

Many of the IDI responses fall into the category of *perceived usefulness*. These findings are grounded in the respondents’ expectations of the CARES app’s potential and their actual experiences in the pilot phase.

#### Usefulness of CARES prototype to improve healthcare delivery.

All respondents stated that the CARES app helps to improve the quality of ART services delivery, from community service delivery preparation through NCAP results reporting. Nurses praised CARES for its practicality in routine tasks, particularly liking features like viral load alerts and automatic client list generation for community visits that helped administer care, especially over the existing ODK tool. They valued CARES’ ability to potentially extract EMRS data directly, saving time spent verifying data. Moreover, some nurses noted that CARES facilitated drug management, providing the necessary quantities needed for community visits. In addition, most nurses expressed fewer data discrepancies due to EMRS data integration.

*“Previously they would do manual work to know which clients are due for viral load. Using the App, they can just generate a list of clients who are due for viral load and that makes their preparation easier and quick….. Even for drugs… this App has the drugs reconciliation per site as such the nurses will be able to know which site has run out of drugs as they are preparing where to pick the drugs, it helps in the drugs reconciliation” (IDI 002)*

#### Usefulness of CARES prototype data to improve data for decision-making.

In addition, nurses emphasized the CARES app’s potential to improve the quality of M&E data, including timeliness, data accuracy, and adherence calculations, mirroring EMRS functionalities. Most mentioned expectations that CARES would enhance real-time clinical decision-making when providing care. Some nurses envisaged that CARES would improve NCAP data management by reducing retrospective entry, offering valuable community data collection, and potentially freeing M&E personnel. M&E respondents echoed these sentiments, emphasizing the app’s role in bridging gaps between community-level data collection and EMRS systems.

*“ It can guide or remind us, let’s take it for instance a client needs to have viral load sample taken or the number of clients that need to be seen in a particular day. It gives us an alert on the clients that we will see…It pops up viral load for those that will be seen “ (IDI 005)*

### Perceived ease of use for CARES prototype (TAM1/ TAM2)

Perceived ease of use is defined as the degree to which a person believes that using a particular system would be free from effort. All IDI respondents reflected perceived ease of use with CARES’ user interface design similar to the EMRS which made it more user-friendly and use of encryption like the EMRS for safer synchronization.

#### Ease of use: CARES prototype to reduce data-related workload.

Respondents highlighted how CARES’ technical advantages made it easier to use than ODK, including better display of information to aid providers during a client visit, enhanced security, and reduced workload. Furthermore, the M&E respondents praised CARES’ design for its compatibility with the EMRS’s data schema, ensuring consistent data collection for M&E purposes and data security due to encryption. More critically, both CARES and M&E respondents highlighted their excitement that CARES was designed to reduce the nurses’ data-related workload. One respondent underscored these expected benefits as there is currently no community-based ART management tool to help reduce the M&E burden.

*“It [my workload] has been reduced …previously the process of printing will take close to two hours, that is just to pull the data and print. This time when they are back, we just pull the data and print within a few minutes” (IDI 007)*

While not all the promise of CARES has been accomplished, all the IDI respondents had high expectations for future CARES’ impact on improving workflow and documentation, providing additional usefulness in future. Most respondents expected a fast and reliable device to streamline NCAP services in the community, explicitly improving data management and making things easier and faster in NCAP service delivery and M&E.

### Output Quality (TAM2)

#### Output quality: Deficiencies in the CARES prototype performance.

Slowness of the app and its tendency to reset dramatically reduced the perceived ease of use, leading to manual data entry and undermining its intended efficiency. All highlighted poor usability issues related to the app’s speed and navigation.

*“We are unable to use the app because it is slow. When I say we end up*

*releasing the client that means we use the old approach where we would*

*capture the information manually and enter EMRS when we are back,*

*as it was before CARES” (IDI 003)*

Poor CARES app speed worsened the nurses’ workload and increased the time spent with the client waiting for the app to load. Respondent IDI 15 complained that “*one of the major frustrations for the nurses, the expectation was that they will be able to see a lot of people within a short period using the app, but it is not helping in that area* “. They also highlighted that the app has not yet achieved integration with the EMRS—a key anticipated feature. As a result, they still have to manually input data into the EMRS.

#### Output quality: issues with consistent data quality.

On the M&E side, the lack of an EMRS sync feature limited the extent of its utilization, increasing rather than decreasing the effort for quality NCAP data. Most nurse respondents noted discrepancies in CARES’ data, including duplicates and client information errors. M&E respondents were concerned with compromising the NCAP database integrity if wrong information was entered into the app. They also reported missing client records, who had to be re-enrolled in the application. In addition, they stated that they need features like daily client schedules and filter options for support groups to ease their workload. As a result, the respondents agreed that CARES is not superior to ODK in its current state, lamenting that the “*CARES app aimed at shortening the time taken to see the client and data entry... but the process is still the same”(IDI 008).*

### Social influence processes (TAM2)

#### Social influence process: Using CARES to meet organizational expectations.

We identified HCWs perceived usefulness of CARES prototype testing to meet organizational expectations as a key, and unique, driver, for CARES acceptability by HCWs. Continued use of CARES was strongly encouraged by organizational leadership who wanted to ensure fulfillment of research objectives and timelines. Healthcare providers’ use of the app, even when it was not performing to expectations, appears driven by the expectations set by the internal research team and the CARES developers. For instance, one nurse reported that due to the app becoming too slow, some nurses chose not to use CARES in the community. However, to meet organization expectations and requirements for CARES testing, they still entered data into the app upon returning to the facility. One IDI noted that they did not use CARES in the community, but still felt obligated to, *“enter the data not for our* [NCAP] *sake but for them [developers] to see that we have entered the data” (IDI 008)*. Forgoing CARES real-time data entry when it lagged in the field and reverting to ODK created subsequent data entry into CARES – a double workload burden for CARES providers and M&E teams that met requests for research needs on top of care delivery.

#### Cognitive instrumental processes: Continued Optimism towards CARES.

Despite the extensive challenges with the CARES prototype, the IDI respondents demonstrated optimism that CARES can be improved for continued and effective use, suggesting that they felt CARES was relevant and potentially helpful for their work. CARES HCWs universally expressed faith in its potential for effective use and wished to continue using the app. Some respondents emphasized the importance of a positive attitude for CARES’ success, highlighting the need for a mindset shift towards accepting the app. They stated that with this change, CARES would thrive. Additionally, the active participation of respondents in the development process indicates their involvement and investment in the app’s success, which can also impact their willingness to continue using it, as noted by IDI 10, who stated, “*We have been so client because we know where we are coming from.”* Some respondents hesitated to express negative opinions about the app because it is still in the beta version, remaining hopeful that further improvements would bring expected gains to fruition.

### Themes beyond TAM models

While the TAM provided a valuable framework for understanding the respondents’ acceptance of the CARES prototype based on perceived usefulness and perceived ease of use, not all themes emerging from the interviews could be neatly categorized within TAM constructs. We identified additional themes that influence the continued use of the CARES prototype, delving into the long-term maintenance and sustainability of CARES.

### Continuous engagement (New)

We observed that continuous engagement is vital to the continued use of CARES. This involves capacity building and engaging key internal and external stakeholders to address challenges and maintain the system.

#### Importance of continuous, iterative HCW inputs into CARES prototype.

Respondents were keen to emphasize that for success, continuous engagement starts internally with Lighthouse Trust and NCAP inputs. The nurses offered extensive comments, feedback, and suggestions on the necessary features to streamline the NCAP process based on their expectations of how CARES app should enhance their work. They shared NCAP SOPs and data collection tools and facilitated developer visits to the community to observe NCAP procedures and grasp NCAP requirements. Nearly all M&E respondents noted their participation in the development process by contributing to the data validation that enhanced the app’s flow and design so that CARES app would strengthen the data management system. During weekly virtual meetings with developers, nurses also noted that they highlighted expected features like viral load reminders and stressed the importance of syncing with the EMRS. This collaborative app development, with users informing features based on their needs, allowed users to offer insights into practical aspects and suggest needed improvements to enhance its utility. Collectively, the respondents felt that the feedback sessions were productive, believing that the app would live up to their expectations.

*We are the ones on the ground and whatever we say they [the developers] would understand and incorporate it. (IDI 011)*

#### Capacity building.

The M&E respondents highlighted the need for comprehensive training and skill transfer for sustainable app maintenance. Nurses added that extended observation for enhancements and broader training, including other cadres such as pharmacists, for effective NCAP support.

[Training for CARES should include] *what is the CARES, what do we want to achieve in terms of the objectives, and what improvements are we intending to achieve by the end of the day. CARES, the providers should understand the objective which is retaining people in care (IDI 002)*

#### Stakeholder engagement.

Respondents noted the important role of stakeholder engagement among key individuals and organizations that are external to the immediate Lighthouse Trust teams. This external collaboration was emphasized primarily by M&E respondents, who stressed the need for ongoing collaboration between CARES developers and MoH’s HIV/AIDS and Digital Health Unit on data related issues. CARES users engaged in discussions with the MoH and other external stakeholders overseeing the EMRS to align the app with national standards and address mHealth gaps. One respondent emphasized the importance of solidifying this technical expertise communication and collaboration, noting:

“*I think we also need some kind of technical expertise on the use of the system, especially when you need pulling and pushing data between EMR and the CARES application” (IDI 009).*

In addition, they deemed concurrence from the MoH, and other key stakeholders involved in the Malawi Health Information System as vital, particularly for integrating CARES with the EMRS.

### Sustainability (New)

Sustainability emerged as a theme that influenced the continued use of CARES. This included a focus on capacity building, stakeholder engagement, infrastructure, data security, and integration with other services.

#### Infrastructure.

IDI respondents underscored the need for resources to maintain CARES, such as tablets for nurses, drug storage, and adequate human resources, as critical for sustainability. Furthermore, the IT manager, stressed the need for proper infrastructure, like servers and support staff, for scaling up.

The data manager suggested appointing an organization like Lighthouse as the app’s custodian if CARES was scaled to other facilities beyond Lighthouse.

*If there could be a deliberate plan that the one who was given the custodian of the application should continue with the system, then it will be good…there is no capacity of government to use or maintain the system. (IDI 009)*

#### Data security.

Respondents unanimously identified data security as paramount to strengthening CARES, advocating for password protection, unique user IDs, and internet access controls in the CARES app. Further suggestions included short data retention within the app, with local clinic storage post-EMRS sync. They also recommended using biometric login, data wipe protocols, clear tablet security responsibilities, and individualized app access to ensure data integrity and prevent misuse.

*It should also have a feature that the moment you make mistakes to access the CARES app in case you have made the mistake on the log in details …then the device should wipe all the data. (IDI 008)*

#### Integration with other services.

When asked about suggestions for expanding CARES prototype features, they proposed enhancements such as monitoring viral load orders, transitioning to online platforms, and adding dashboards to expand CARES functionality. Some nurses recommended expanding the app’s services to incorporate other healthcare services, such as cervical cancer screening and non-communicable diseases.

*If clients are already available in the app, then they can bring a scanner instead of searching for the client, we can scan the barcode and it will be able to bring the client, that will help to reduce the amount of time. (IDI 006)*

The integration of CARES with other mHealth interventions, notably the Two-way Texting (2wT) SMS delivery system currently piloted at Lighthouse, was also recommended by some M&E respondents.

*Maybe if it could be integrated with their phones, maybe getting the reminder direct from the CARES to the client, the appointment, getting medication, motivation…Just as the way it is with two-way texting. (IDI 009)*

## Discussion

This study analyzed healthcare providers’ perspectives on the feasibility and acceptability of the mHealth CARES prototype, examining its role in facilitating NCAP services for clients, nurses, and M&E teams. We identified important factors facilitating HCWs’ acceptance of mHealth technologies—healthcare delivery improvement, meeting organizational expectations, collaborative development using HCD, and sustainability initiatives. We also documented formidable barriers to CARES success, including app performance issues, data quality discrepancies, navigation challenges, data security concerns, infrastructure, and resource limitations. We framed our results based on the extended TAM2 [30] theoretical framework that explores perceived usefulness and perceived ease of use while providing insights into a CARES optimization pathway for effective and continued use. We added the additional themes - stakeholder engagement and sustainability – that might support future adoption efforts if other optimization is achieved. We discuss how these results informed additional CARES iterations, improvements, and plans for future adaptations.

First, applying the TAM2 framework helped underscore the influence of perceived usefulness, output quality, social process influences, and ease of use in CARES technology acceptance. HCWs anticipated the CARES prototype would enhance efficiency, aligning with TAM2’s cognitive instrumental process [30]. We noted user expectations and experiences with technological improvements as a key factor shaping HCWs’ perceptions of CARES. Their prior experiences with ODK and EMRS influenced how they assessed CARES’ potential benefits and shortcomings. They praised the ability of the CARES app to streamline tasks such as community visit preparations and drug management, indicating high perceived usefulness.

However, while HCWs expected improvements in efficiency, deficiencies in output quality, a key TAM2 construct reflecting technology performance [30], hampered usability and reduced its intended benefits due to the app’s slow speed, system resets, and data discrepancies. Additionally, the concept of subjective norm within TAM2 [30] reflects the societal influences on an individual’s decision to use a technology. Providers continued using CARES amidst greater workloads, showing their commitment during its prototype phase and underscoring the role of stakeholder expectations in technology adoption decisions. It suggests that when HCWs believe in the importance of the specific technology to their supervisor’s or the organization’s goals, they may be more willing to adapt their work practices to incorporate the technology at the development stage.

While TAM2 provided a strong foundation for analyzing technology adoption, newer extensions such as TAM3 [35] introduce additional determinants, including experience as a moderator and intervention-based influences. Though our study was guided by TAM2, the evolving nature of TAM models suggests the importance of continued adaptations to reflect the complexities and rapidly-evolving landscape of mHealth implementation in low-resource settings.

Beyond the TAM frameworks, we identified user optimism, continuous engagement, and sustainability as crucial aspects of CARES feasibility for continued and effective use, confirming past research that links HCW attitudes and stakeholder commitment to mHealth success [36–38]. On the positive side, providers accepted many of the current CARES weaknesses and remained optimistic about continuing to use CARES if improvements were attained. Nurse users emphasized the potential for a positive impact on client care, while M&E respondents focused on technical and programmatic considerations. These improvements could increase healthcare quality as a result of the mHealth tool, a facilitator of mHealth implementation success, as seen previously [36–39]. However, similar to the findings from a systematic review of HCW acceptability of mHealth interventions, we found that continued issues in CARES technical failures and resulting increased workload were significant barriers to CARES, curbing enthusiasm [40]. HCWs reported frustration about the CARES prototype’s slow performance and difficulties with EMRS synchronization, again decreasing engagement and buy-in. HCW users cited the unfilled promise of improved workflow, streamlined data management, and overall efficiency for NCAP service delivery but also suggested that overcoming these issues would further positive attitudes toward future CARES interactions. Lastly, sustainability, which centered around critical improvements in infrastructure, connectivity, data security, and technical support, emerged as essential to scaling up and sustaining mHealth intervention. Sustainability appeared tied to a strong commitment by both HCWs and relevant stakeholders, as seen elsewhere in mHealth expansion success regionally [41,42].

The HCD process appeared crucial for HCWs’ acceptance and buy-in. Their involvement in the design phase, sustained through an 18-month engagement alongside their duties, reflects an enormous dedication to NCAP clients and the improvement of care delivery. Moreover, their consistent inputs for iterative testing helped increase CARES‘prototype tailoring to the Malawian context and the unique needs of NCAP and the EMRS at Lighthouse. This approach also secured organizational backing, addressing a common barrier in mHealth implementation [38,43]. These issues necessitated supplementary documentation methods, raising questions about the app’s practicality and, thus, the usefulness of the app, even if they recognize its theoretical benefits [44,45]. Technological challenges, which are common barriers to implementing and adopting mHealth interventions [41,46,47] were among the factors contributing to the delays in addressing CARES issues.

Our research underscores that while TAM2 provides a robust framework for understanding the key determinants of technology acceptance, it must be adapted and contextualized within healthcare professionals’ specific operational workflows and user experiences. We excluded behavioral intention from our model because, in our study, participants were interviewed after already experiencing the intervention, describing perceptions of actual usage rather than intentions. Also, previous research on technology acceptance showed that behavioral intention becomes a less significant predictor of actual use when the likelihood of use is initially high [48]. However, we also included the continued use of CARES in our model to explain if and how HCWs will continue using CARES in the future.

Our findings highlight the importance of user-centered design in healthcare app adoption and efficacy where HCW and stakeholders are involved in every step—from design to iterative testing—to tailor the app to their actual needs and preferences. Additionally, securing organizational support and aligning the app with institutional objectives encourages healthcare workers to seamlessly incorporate new technology into their daily workflow.

HCWs are optimistic about CARES’ potential to reduce their workload if output quality is improved. If implemented effectively, CARES could also shorten client visit time spent on data collection, enhancing the benefits of NCAP for clients. Integrating CARES with the EMRS is critical to maximizing its impact on reducing workload. However, further development is needed to implement this function, requiring continued collaboration with HCWs to ensure it is effective and does not inadvertently increase workload.

The study has several strengths and limitations. Among strengths, we show that consistent involvement of HCWs who will use an mHealth platform should be central to the design, development, implementation, and evaluation of that mHealth tool. This HCD approach should be required for mHealth to ensure right fit technology for its intended purpose. Second, we demonstrate that application of behavioral theory can help guide understanding and provide insights into mHealth implementation, synthesizing user insights for further optimization. For limitations, the sample size was small but included all NCAP providers who used CARES, an actuality that should be acknowledged. Secondly, the evaluation occurred while the app was still undergoing testing and enhancements; interviews were not repeated after further improvements in technology and usability. Third, the interviewers’ affiliation with Lighthouse Trust and familiarity with NCAP may have influenced participant responses, a social desirability bias that may have influenced participants’ responses in either positive or negative directions towards CARES.

### Conclusion

Overall, HCWs largely embraced the promise of the CARES prototype even while recognizing that the reality of current CARES falls short. High levels of HCW involvement in the app’s development likely facilitated their commitment to CARES success. The insights obtained from these interviews offered guidance for refining the CARES app, and these recommendations are being systematically addressed through iterative CARES improvements. If incorporated successfully, continued user optimism for the impact of the CARES app to streamline their work and improve client care will be rewarded. In contrast, if the CARES prototype is not enhanced for a smoother user experience in the community and clinic, and integration with the EMRS is not achieved to streamline the data delivery workload, there is a high risk of discontinuation. Continued collaboration among developers, NCAP providers, and other stakeholders, paired with sufficient financial investment in CARES improvement, is the essential next step. Such efforts will facilitate longitudinal assessments to evaluate CARES’ efficiency in reducing HCW workload, its impact on client outcomes, and its role in strengthening NCAP. With that support, CARES can realize its potential to extend the reach of the EMRS into community settings, ensuring that clients in clinic and community settings receive high-quality, integrated care.

## Supporting information

S1 Appendix COREQ (COnsolidated criteria for REporting Qualitative research) checklist. (DOCX)

S2 Appendix Interview Guide. (DOCX)
