# Supplementary material for: Acceptability and feasibility of a mobile electronic medical record system for community-based antiretroviral therapy in Lilongwe, Malawi: A rapid qualitative analysis
Source: PLoS One. 2025 May 23;20(5):e0303416. doi: 10.1371/journal.pone.0303416 (PMC12101664; doi:10.1371/journal.pone.0303416)
Supplement: S1 Appendix — (DOCX) [file pone.0303416.s001.docx]

# **Appendix 1: COnsolidated criteria for REporting Qualitative research (COREQ) Checklist [1]**

| **No** | **Item** | **Guide questions/description** | **Reported on Page no.** |
| --- | --- | --- | --- |
| **Domain 1: Research team and reflexivity** | | | |
| Personal Characteristics | | | |
| 1 | Interviewer/facilitator | Which author/s conducted the interview or focus group? | P.11 “All interviews were run by an external, experienced qualitative female researcher using a semi-structured interview guide.” |
| 2 | Credentials | What were the researcher's credentials? *E.g. PhD, MD* |  |
| 3 | Occupation | What was their occupation at the time of the study? |  |
| 4 | Gender | Was the researcher male or female? |  |
| 5 | Experience and training | What experience or training did the researcher have? |  |
| Relationship with participants | | | |
| 6 | Relationship established | Was a relationship established prior to study commencement? | P.11 “The interviewer is an employee of LT, but was not involved with CARES. The interviewer was interested in our research and previously worked in NCAP research.” |
| 7 | Participant knowledge of the interviewer | What did the participants know about the researcher? e*.g. personal goals, reasons for doing the research* |  |
| 8 | Interviewer characteristics | What characteristics were reported about the interviewer/facilitator? e.g. *Bias, assumptions, reasons and interests in the research topic* |  |
| **Domain 2: study design** | | | |
| Theoretical framework | | | |
| 9 | Methodological orientation and Theory | What methodological orientation was stated to underpin the study? *e.g. grounded theory, discourse analysis, ethnography, phenomenology, content analysis* | P. 8 “Analysis of IDIs was guided by the extended Technology Acceptance Model (TAM2)” |
| Participant selection | | | |
| 10 | Sampling | How were participants selected? *e.g. purposive, convenience, consecutive, snowball* | P.7 “We conducted in-depth interviews (IDIs) of HCWs who used the CARES prototype … and those who worked on the technical side, managing, and processing the app’s data” |
| 11 | Method of approach | How were participants approached? e*.g. face-to-face, telephone, mail, email* | P.7 “All HCW involved with NCAP were approached to participate in the interviews” |
| 12 | Sample size | How many participants were in the study? | P.12 “We interviewed 15 HCWs involved with NCAP..“ |
| 13 | Non-participation | How many people refused to participate or dropped out? Reasons? | P.7 “All proposed IDI participants … agreed to participate in the study” |
| Setting | | | |
| 14 | Setting of data collection | Where was the data collected? e*.g. home, clinic, workplace* | P.10 “Data was collected at LT clinic” |
| 15 | Presence of non-participants | Was anyone else present besides the participants and researchers? | P.11 “No one else was present in the interviews besides the interviewer and respondents” |
| 16 | Description of sample | What are the important characteristics of the sample? *e.g. demographic data, date* | P.12 “The majority were male (N=9) and nurses (N=10). The mean age of the participants was 38, with an average duration of employment at Lighthouse and involvement with the NCAP being seven years and four years, respectively. The interviews lasted, on average, 34 minutes.” |
| Data collection | | | |
| 17 | Interview guide | Were questions, prompts, guides provided by the authors? Was it pilot tested? | P.11 “All interviews … using a semi-structured interview guide“ |
| 18 | Repeat interviews | Were repeat interviews carried out? If yes, how many? | P.11 “None of the interviews were repeated.” |
| 19 | Audio/visual recording | Did the research use audio or visual recording to collect the data? | P.11 “. IDIs were..recorded and transcribed verbatim.” |
| 20 | Field notes | Were field notes made during and/or after the interview or focus group? | P.11 “the interviewer recorded field notes during select interviews. |
| 21 | Duration | What was the duration of the interviews or focus group? | P.12 “The interviews lasted, on average, 34 minutes.“ |
| 22 | Data saturation | Was data saturation discussed? | P.7 “By interviewing all HCWs involved in the NCAP program, we anticipated reaching a natural point of data saturation.” |
| 23 | Transcripts returned | Were transcripts returned to participants for comment and/or correction? | P.11 “The transcripts were not returned to the participants.” |
| **Domain 3: analysis and findings** | | | |
| Data analysis | | | |
| 24 | Number of data coders | How many data coders coded the data? | P.11 “Two researchers initially summarized the first three transcripts” |
| 25 | Description of the coding tree | Did authors provide a description of the coding tree? | P.11 “Instead of employing a traditional coding tree, the interview transcripts were condensed into summary templates“ |
| 26 | Derivation of themes | Were themes identified in advance or derived from the data? | P.11 “Insights were discussed until a consensus was reached on the themes and sub-themes” |
| 27 | Software | What software, if applicable, was used to manage the data? | P.11 “…the content of the summaries for each domain into an Excel file” |
| 28 | Participant checking | Did participants provide feedback on the findings? | P.11 “The study participants did not provide feedback on the findings.” |
| Reporting | | | |
| 29 | Quotations presented | Were participant quotations presented to illustrate the themes / findings? Was each quotation identified? e*.g. participant number* | P.13-23 in the results section |
| 30 | Data and findings consistent | Was there consistency between the data presented and the findings? |  |
| 31 | Clarity of major themes | Were major themes clearly presented in the findings? |  |
| 32 | Clarity of minor themes | Is there a description of diverse cases or discussion of minor themes? |  |

[1] Tong A, Sainsbury P, Craig J. Consolidated criteria for reporting qualitative research (COREQ): a 32-item checklist for interviews and focus groups. International Journal for Quality in Health Care 2007;19:349–57. https://doi.org/10.1093/intqhc/mzm042.
