# Supplementary material for: Acceptability and feasibility of a mobile electronic medical record system for community-based antiretroviral therapy in Lilongwe, Malawi: A rapid qualitative analysis
Source: PLoS One. 2025 May 23;20(5):e0303416. doi: 10.1371/journal.pone.0303416 (PMC12101664; doi:10.1371/journal.pone.0303416)
Supplement: S2 Appendix — (DOCX) [file pone.0303416.s002.docx]

**Interview of Healthcare workers involved in CARES**

FIRST: Interviewer MUST verify that informed consent is signed by the interviewee

1. Verification of informed consent? CIRCLE ONE: YES NO

General introduction:

Hello, my name is ____. Thank you for agreeing to talk to me today. I am here to talk with you as part of Lighthouse Trust. We recently launched the CARES mobile electronic medical records app here in NCAP settings to see if we could increase the quality of care in the community setting. Then, we upload the CARES data to the clinic EMR to ease monitoring and evaluation of patient data. So far, we are collecting NCAP data on the CARES App instead of using the previous ODK system for Lighthouse and MPC clients, only. We hope to expand to other NCAP settings and Lighthouse satellite clinics over time.

I am here to talk to you about your thoughts, opinions, and suggestions about the CARES design, development, and launch process. You are being asked to this interview as you participated in this process in NCAP and/or in this clinic. As a staff member, your experience and thoughts about CARES are critical to our understanding of the CARES app and how to make it better. You may also choose not to answer any question. If a question is unclear to you, you can ask me to explain it. Your participation is voluntary and confidential. Whatever you tell me will be treated with utmost confidentiality. The information will only be used for the purposes of this program evaluation.

I would like to request that you all allow me to record our discussion so that I don’t miss anything. Your voice will not be heard by anyone other than the people here and our study transcriber. Your name will not be recorded and will not appear on the transcription. The tapes will be destroyed after we have prepared our transcripts.

Is it okay if I tape record our discussion? YES NO [IF NO, STOP]

1. “To begin, tell me your role in NCAP or the NCAP M&E process, including your position and responsibilities.”
2. Now, let’s talk about CARES, specifically. Let’s start with the **design** (format, forms, features) of the CARES app, itself.
   1. Tell me about your role in App design – BEFORE the launch (getting the right questions and format into CARES)?
   2. How was your feedback included in App iterations BEFORE launch?
   3. What worked well in this phase?
   4. What was challenging in this phase?
3. Let’s talk about using CARES **in the field with clients or with client data TODAY or currently**.
   1. Tell me about your training to use the app?
      1. How could it be improved?
   2. Tell me about your experience using CARES for clients or client data?
   3. Let’s start with the benefits you see now. What are the **current strengths of using CARES**?
      - 1. For Client care?
           1. Probe: before heading to NCAP sites?
           2. Probe: in the community at NCAP sites?
           3. Probe: when returning from NCAP sites?
        2. For data management?
           1. Probe: before heading to NCAP sites?
           2. Probe: in the community at NCAP sites?
           3. Probe: when returning from NCAP sites?
        3. How does CARES reduce your workload?
           1. Probe: before heading to NCAP sites?
           2. Probe: in the community at NCAP sites?
           3. Probe: when returning from NCAP sites?
   4. Now let’s discuss the problems you see now. What are the **current problems and pitfalls of using CARES**?
      - 1. For Client care/management?
           1. Probe: before heading to NCAP sites?
           2. Probe: when returning from NCAP sites?
        2. For data management?
           1. Probe: before heading to NCAP sites?
           2. Probe: when returning from NCAP sites?
        3. How does CARES add to your workload?
   5. Would you consider current CARES a success or a failure? Why?
   6. Would you recommend CARES to other providers or sites? Why?
4. Let’s talk about making CARES better in the near future.
   1. For clients, what CARES features are needed to improve NCAP client care?
   2. For Nurses, what features or functions can help reduce nurse workload?
   3. For M&E teams/data managers, what features or functions could reduce M&E data workload?
   4. How can we improve data security and privacy in the CARES app?
5. Let’s talk about bringing CARES to scale across NCAP sites or in Malawi.
   1. What features or functions could make CARES a success?
      1. For clients
      2. For nurses
      3. For M&E teams
   2. What would make CARES fail?
      1. For clients?
      2. For nurses?
      3. For M&E?
   3. Using your own experience, what do you think the training needs would be to scale CARES?
6. What can we do to make CARES a success in the future?
   1. What challenges should we anticipate?
      1. Probe: EMRs integration?
      2. Probe: maintenance of the app?
   2. How can we best overcome these challenges?
   3. What would “success” look like for CARES expansion?
7. Lastly, thinking just about NCAP, how could NCAP, the program, be improved?
   1. Is there a different app or data support tool that could support these improvements?

END OF SESSION: “Now we have come to the end of our discussion. **Is there anything you would like to add that you haven’t mentioned yet?** Thank you!
